# The hidden biodiversity knowledge split in biological collections

**DOI:** 10.1098/rspb.2025.1045

**Published:** 2025-11-05

**Authors:** Gabriel Nakamura, Bruno Henrique Mioto Stabile, Livia Estéfane Fernandes Frateles, Matheus Lima Araujo, Emanuel Bruno Neuhaus, Manoela Maria Ferreira Marinho, Melina de Souza Leite, Aline Richter, Liuyong Ding, Tiago Magalhães da Silva Freitas, Bruno Soares, Weferson Júnio da Graça, Mario R. Moura, José Alexandre Felizola Diniz-Filho

**Affiliations:** ^1^ Department of Genetics, Ecology and Evolution, Universidade Federal de Minas Gerais, Belo Horizonte, Minas Gerais, Brazil; ^2^ Universidade Estadual de Maringa, Maringá, Paraná, Brazil; ^3^ Programa de pós-graduação em Ecologia e Evolução, Universidade Federal de Goias, Goiânia, Goiás, Brazil; ^4^ Universidade Federal de Goiás, Goiânia, Brazil; ^5^ Instituto Tecnologico Vale Desenvolvimento Sustentavel, Belém, Pará, Brazil; ^6^ Biosciences Institute, Universidade Federal de Mato Grosso do Sul, Campo Grande, Mato Grosso do Sul, Brazil; ^7^ Departament of Ecology, Universidade de Sao Paulo, São Paulo, Brazil; ^8^ Centro Nacional de Conservação de Répteis e Anfíbios, Instituto Chico Mendes de Conservacao da Biodiversidade, Brasília, DF, Brazil; ^9^ Yunnan Key Laboratory International Rivers and Transboundary Eco-Secoruty, Yunnan University, Kunming, Yunnan, People’s Republic of China; ^10^ Institute of International Rivers and Eco-security, Yunnan University, Kunming, Yunnan, People’s Republic of China; ^11^ Universidade Federal do Pará—Campus Breves, Breves, Brazil; ^12^ Institute of Environmental Change & Soci, University of Regina, Regina, UK; ^13^ Universidade Estadual de Maringá, Maringá, Paraná, Brazil; ^14^ Departamento de Sistemática e Ecologia, Universidade Federal da Paraiba, João Pessoa, Paraiba, Brazil; ^15^ Departamento de Biologia Animal, Universidade Estadual de Campinas, Campinas, Sao Paulo, Brazil; ^16^ Departamento de Ecologia, ICB, Universidade Federal de Goiás, Goiânia, Goiás, Brazil

**Keywords:** name bearers, ichthyological collections, reference material, taxonomy, colonial legacy, knowledge distribution

## Abstract

Ecological and evolutionary processes generate biodiversity, yet how biodiversity data are organized and shared globally can shape our understanding of these processes. We show that name-bearing type specimens—the primary reference for species identity—of all freshwater and brackish fish species are predominantly housed in Global North museums, disconnected from their countries of origin. This geographical divide creates a ‘knowledge split’ with consequences for biodiversity science, particularly in the Global South, where researchers face barriers in studying native species’ name bearers housed abroad. Meanwhile, Global North collections remain flooded with non-native name bearers. We relate this imbalance to historical and socioeconomic factors, which ultimately restrict access to critical taxonomic reference materials and hinder global species documentation. To address this disparity, we call for international initiatives to promote fairer access to biological knowledge, including specimen repatriation, improved accessibility protocols for researchers in countries where specimens originated and inclusive research partnerships.

## 1. Introduction

Museums can be viewed as gatekeepers of representative fragments of the world. They provide a valuable source and representation of human history, culture, knowledge and biodiversity. Natural history museums and biological collections (hereafter referred to as biological collections) are known for maintaining an organized and curated record of extant and extinct specimens. Given this role of biological collections, numerous authors have highlighted various ways in which they enhance our understanding of the natural world by shedding light on global change phenomena and spatial and temporal ecological processes [1], public health [2] and educational purposes [2]. Despite their many applications, the primary role of biological collections remains their significance in taxonomic research—particularly in a context in which approximately 80% of extant species have yet to be formally described [3,4]. The discovery of these species often depends on comparing potential new taxa with representative specimens of already-known species preserved in biological collections.

Among the specimens in biological collections, the name-bearing types (hereafter ‘name bearers’) hold special importance in biodiversity studies (box 1). Name bearers are pivotal in taxonomic studies, as they constitute the fundamental reference on which taxonomists rely to review described species or propose new ones [6,7]. The accessibility of name bearers is therefore crucial, as the most accurate assessment of certain taxonomically relevant features requires direct examination of the original reference specimen. While the digitization of name bearers and modern molecular techniques for species identification (e.g. DNA barcoding) are undeniably important for biodiversity studies [8], the original material cannot be replaced solely by indirect data or molecular information for most organisms [9]. For example, around 562 tetrapod species have not been observed in over 50 years, and at least 40% are known only from the holotype [10]. The irreplaceable nature of these holotypes underscores their importance for conservation and taxonomic research, as their absence in biological collections cannot easily be compensated for through field observations.

Box 1.
**A glimpse into taxonomic nomenclature**

Name-bearing types (or name bearers) are specimens, or sets of specimens, designated as the standard reference for applying the name they bear. Each nominal taxon has an actual or potential name bearer. The objective of this nomenclatural act provided and regulated by the International Commission on Zoological Nomenclature (ICZN) is to fix a name in an international standard of reference, bringing stability to the name usage of a nominal taxon [5]. Four distinct types of name bearer are applied to fish in different cases, as shown in the figure below.

**Figure:**
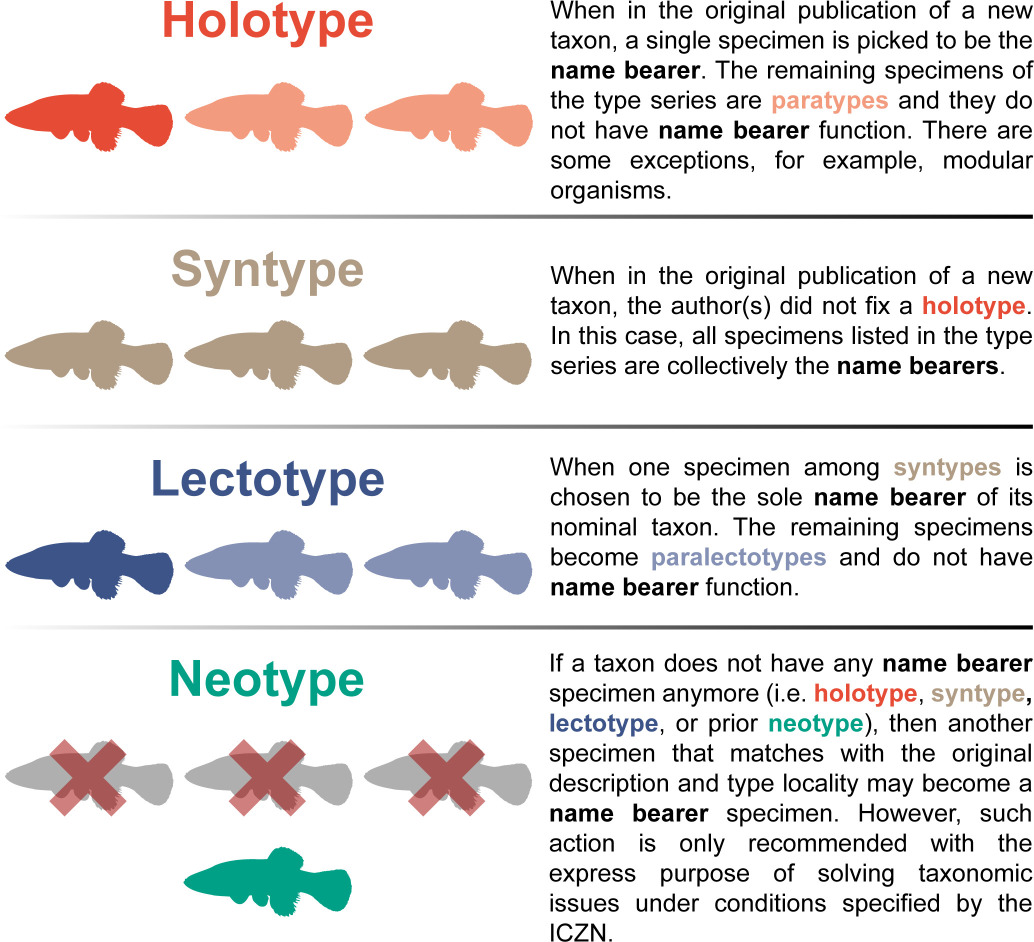

Recently, palaeontology has drawn the scientific community’s attention to the importance of name bearer distribution in biological collections (e.g. see the *Ubirajara* case [11]). Given the rarity of palaeontological records that can be used to identify a species, this unique sample often serves as the name bearer for a given species. In this context, recent studies have shown that a biased concentration of fossil samples in biological collections located in the Global North (countries with developed economies and a significant influence on global environmental governance, often located in the Northern Hemisphere) profoundly affects our view of deep-time biodiversity [12]. Beyond the impacts on our understanding of nature, this knowledge concentration also jeopardizes educational initiatives and scientific development in certain nations, as much of the research effort and public interest in biological collections depends on the presence of these materials [13]. While biases in name bearer distribution in palaeontology are receiving more attention from the scientific community and the public, similar issues extend to extant organisms, producing significant consequences for biodiversity science—especially in the context of accelerated biodiversity loss [14]—with more negative effects experienced by countries from the Global South.

Like other forms of biological data, the representation, curation and concentration of name bearers in biological collections worldwide are subject to biases, which can hinder accessibility—particularly for researchers based in countries where most of their native species are housed abroad. Taxonomists frequently report that many name bearers, particularly from the tropics, were collected and sent abroad, mainly to biological collections in Europe and North America during the late eighteenth and nineteenth centuries [15,16]. For instance, less than 18% of name-bearing reptile types are housed in Global South collections [17], despite this region harbouring 81% of reptile species [18]. Moreover, the number of plant species preserved in biological collections is twice as high in some Global North countries [19]. This colonial legacy in species name bearers can hinder efforts to accurately catalogue and organize knowledge of biological diversity [20]. Since the concentration of name bearers in certain countries reflects historical socioeconomic dynamics, a country’s economic power has played—and continues to play—a crucial role in shaping the accumulation of biodiversity collections.

We argue that a fairer distribution of name bearers is necessary to close biodiversity knowledge gaps. In this study, we mapped the origin and destination of name bearers over time to identify macropolitical imprints in biodiversity knowledge accumulation. This study explores how biodiversity knowledge accumulates and shapes our perception of the natural world. We focused on freshwater and brackish fishes, the most species-rich group of vertebrates, which face challenges from uncertainties in their evolutionary relationships and ongoing taxonomic changes, making name bearer assessment crucial for improving accessibility of biodiversity knowledge [21]. We address the following questions. Where are name bearers located globally? How have countries accumulated biodiversity knowledge over time? Ultimately, we provide solutions and best practices to address these questions.

## 2. Methods

### (a) Data acquisition

We compiled all freshwater and brackish fish species names using the Eschmeyer’s Catalog of Fishes (CAS) [22], the most updated curated taxonomic database for fishes. We extracted 15 303 valid names and 4943 synonyms. We included the synonyms because they also correspond to reference specimens in biological collections recognized as name-bearing types. Additionally, they are essential for conducting taxonomic reviews of species that were previously classified under these names. This resulted in 20 246 binomials that we called name bearers, following the definition of the International Code of Zoological Nomenclature [5]. For each name bearer, we extracted the country where the specimen (or specimens in case of syntype) was collected (i.e. the source country) and the museum housing the respective name bearer. Based on the abbreviations of the biological collections in CAS , we obtained the country where each museum/biological collection is located. If the source country of the name bearer was not specified, we checked CAS for additional information, such as coordinates or any other geographical location. We excluded marine species from our database due to the challenges associated with defining their distribution within political boundaries. All the searches in CAS were performed between March 2023 and May 2024.

### (b) Spatial characterization of name bearers across time

**Figure 1.**
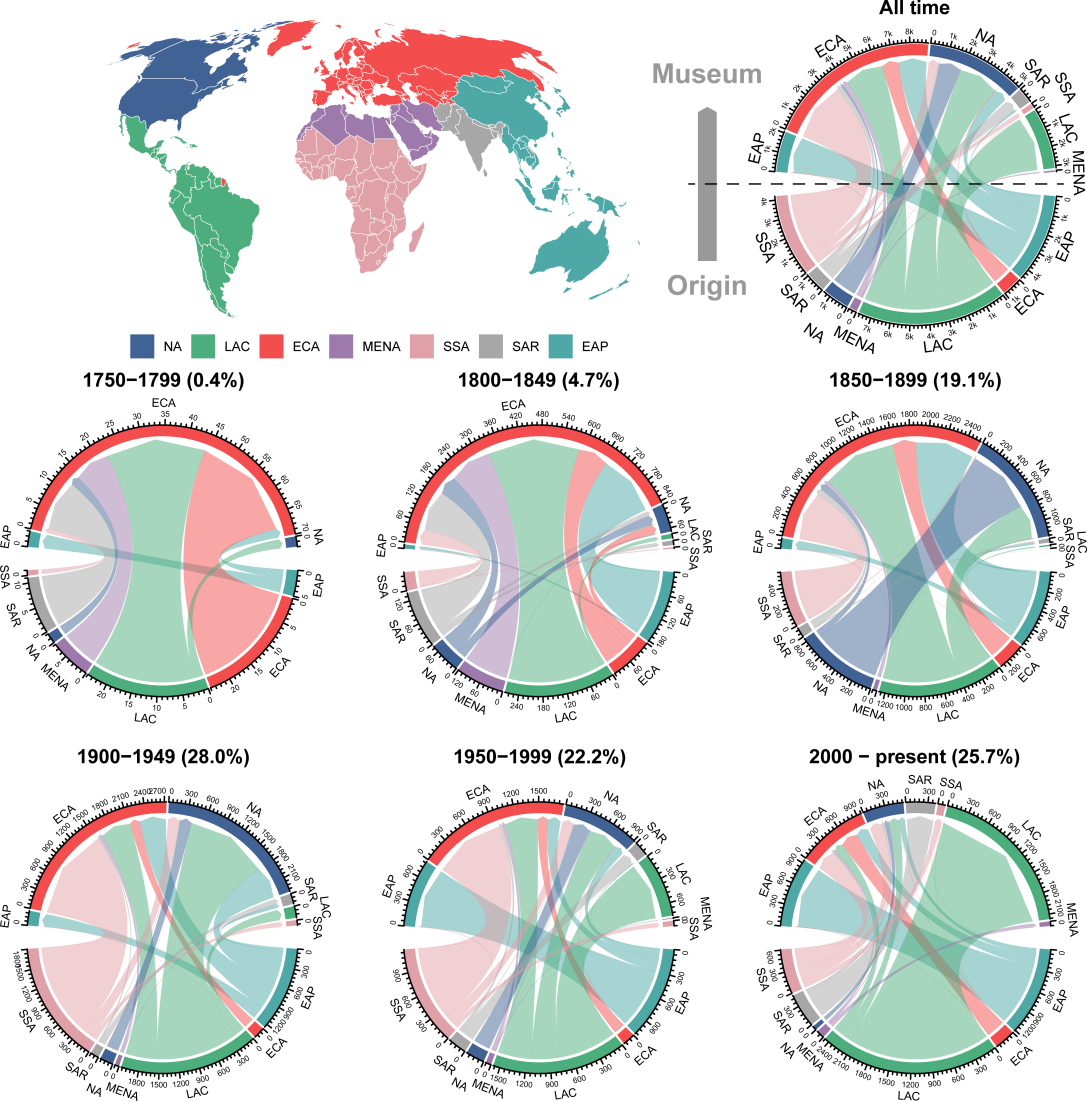
Global flow of fish name-bearing specimens among macropolitical regions in 50-year time intervals. Across all chord diagrams, macropolitical regions are coloured according to World Bank classification as illustrated in the upper left cartogram. The lower half of the circular plots represents the regions, where the name bearer was collected (source regions), and the upper half is the region of the biological collection in which the name bearer is housed (housing regions). The numbers on the outer circles represent the total number of name bearers exchanged between each region in each period. The percentage besides the year corresponds to the percentage of name bearers of that period relative to the total name bearers in all periods. Macropolitical regions: ECA, Europe and Central Asia; NA, North America; SSA, Sub-Saharan Africa; SAR, South Asian Region; MENA, Middle East and North Africa; LAC, Latin America and the Caribbean; EAP, East Asia and Pacific. The red region within LAC corresponds to French Guiana, an overseas territory of France, and is thus categorized as part of ECA in the map.

We mapped the country where each name bearer was collected (source country) and its current housing country. With this information, we characterized the flow of name bearers in 50-year intervals, grouping the countries according to the World Bank classification obtained from R package *countrycode* [23] (figure 1). We opted for the World Bank classification of countries, as it more accurately represents global regions based on overall infrastructure and economic characteristics, which we consider crucial in determining the distribution of name bearers. For example, some Latin American countries that are more similar in terms of infrastructure and economy are grouped with countries with very different economic contexts under other geographical classifications (e.g. Mexico grouped with USA and Canada under other classifications). The origin and destination of name bearers were used to derive two metrics that relate to the native composition of each country, and its name bearer composition housed in their biological collections. These two metrics are explained in the section below.

### (c) Congruence between name bearers and endemic fish composition

Biological collections should ideally house name bearers from the country’s ecological and geographical context to maximize biodiversity benefits [13], such as for biological research and educational purposes. Here, we used two metrics to assess how well a country’s name bearer collections reflect its endemic species composition (per country native species richness is shown in electronic supplementary material, figure S1). Endemic species composition was extracted directly from CAS. Here, we classified endemic name bearers as those species with geographic distribution (according to CAS) restricted to a single country. Further information on species composition data and analysis with all native species per country is presented in electronic supplementary material (richness and country species composition from CAS).

The first metric, endemic deficit, is the proportion of the known endemic species lacking in a country’s collections (*endemic deficit* = *number of endemic name bearers outside of the country of origin* ÷ *country’s endemic species richness*). High values of this metric indicate countries missing most of their endemic species’ composition in their biological collections. The second metric measured the proportion of country’s name bearers that belong to non-native species (*non-endemic representation* = *number of non-endemic name bearers* ÷ *country’s endemic name bearer richness*). Higher values identify countries with an overrepresentation of endemic species from other countries.

We decided to present here only the analysis with endemic species, because it is a more conservative way to show the mismatch between native fish fauna and the name bearers housed in biological collections. This filter ensures that we did not underestimate the representation-based metrics, where wide-ranging species were first sampled in foreign countries. We also calculated the representation-based metrics for the full dataset (see electronic supplementary material, figure S3), but the results were qualitatively similar with only minor quantitative differences.

### (d) Determinants of per-country number of name bearers and related metrics

We investigated five factors potentially driving the per-country number of name bearers: (i) per-country number of native fish species; (ii) per-country number of preserved fish specimens extracted from Global Biodiversity Information Facility (GBIF), corrected by the respective country’s area; (iii) per-country gross domestic product (GDP); (iv) per-country number of ichthyological collections; and (v) number of years since independence for formerly colonized countries (for countries that never experienced colonization the value of this metric is zero). All explanatory variables were centred and scaled (*z*-transformed) to allow direct comparisons of their effect sizes [24]. We then used a generalized linear model (GLM) with negative binomial error distribution to estimate the per-country number of name bearers.

We also checked whether these five explanatory variables affected the country-level metrics (endemic deficit and non-native representation) using GLMs with a beta-binomial error distribution [25]. To assess the validity of our models, we evaluated the homoscedasticity in the residuals against predicted values (model assessment graphics are available in electronic supplementary material, figures S3) and through qq-plots using a simulation approach [26]. The standardized effect sizes for all explanatory variables are provided in electronic supplementary material, tables S1–S5.

### (e) Data sources

The primary data used in this article were taken from Eschemeyer’s CAS [22]. CAS also provided information on name bearers sampling location, museums, native species distribution and the number of museums. Bio-Dem (https://bio-dem.surge.sh) provided socio-economic variables (GDP, years since independence). GBIF provided the number of occurrences per area. The number of museums, number of native species per country, GDP, years since independence and number of occurrences per area according to GBIF were used in the modelling approach.

## 3. Results

### (a) Spatial characterization of name bearers across time

Since 1758, a total of 20 246 fish binomials have been described globally. Of these binomials, 13 325 name bearers were collected in countries within the Global South (Latin America and Caribbean, Sub-Saharan Africa, Middle East and North Africa and South Asia), compared with 6921 name bearers collected in countries of the Global North (North America, Europe and Central Asia and East Asia and Pacific). Notably, although a greater number of name bearers were sampled in the Global South, these regions are home to only 4208 name bearers, whereas all Global North countries collectively house 16 038, corresponding to 79% of all freshwater name bearers.

Museums in Europe and Central Asia (ECA) and North America (NA) have historically served as the primary destination for freshwater/brackish name bearers from all continents, with their dominance in housing name bearers peaking between 1900 and 1949, when nearly 90% of name bearers (*n* = 5051) were housed in these regions (figure 1). Only in the past two decades there has been a trend for countries to retain their name-bearing specimens within their own ichthyological collections (figure 1, 2000–present). Although recent patterns in name bearer retention can be related to the establishment of Global South biological collections in the last 50 years, Global North biological collections still keep the historical pattern of housing foreign name bearers. For instance, 37% of the 2466 name bearers housed in biological collections of the Global North (East Asia and Pacific, Europe and Central Asia and North America) during the 2000s were representatives of fish faunas from the Global South (Latin America and Caribbean, Middle East and North Africa, South Asia and Sub-Saharan Africa), with the opposite occurring for 0.2% (6 name bearers out of 2732) of name bearers housed in the Global South countries.

### (b) Congruence between native fish composition and name bearers

**Figure 2.**
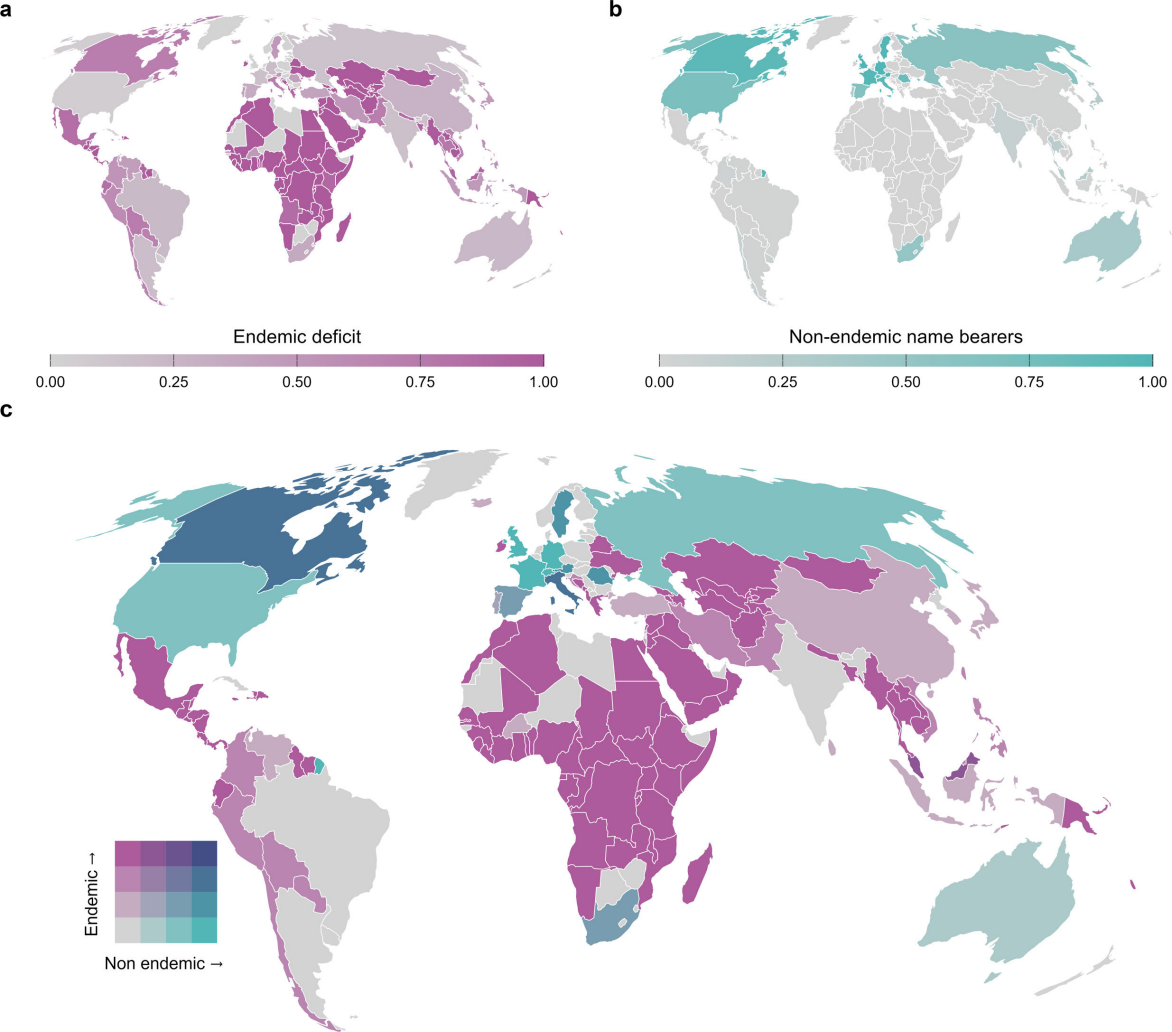
Global patterns in the representation of endemic and non-endemic fish name bearers across countries’ ichthyological collections. (a) Endemic deficit, as the proportion of the known endemic species lacking in a country’s collections. (b) Non-endemic name bearers, as the proportion of country’s name bearers that belong to non-native species. (c) Both mapped patterns combined in a bivariate map (c), where the breaks represent data quartiles (0–25%, 25–50%, 50–75% and 75–100%). See the main text for the definition of endemic species.

The endemic deficit was very low for some countries in the Global North (e.g. 5% for USA, 15% for France, 20% for Germany and 0% for Great Britain). On the other hand, this proportion was high for most Global South countries, with a mean of 88% of endemic deficit, with mega-diverse countries having high values, such as Ecuador (81%), Bolivia (72%) and Benin (100%). Countries in Europe and Central Asia housed the majority of name bearers representing their endemic fish fauna, while collections in South Asia and Middle East and North Africa lacked the name bearers for most of their endemic species (figure 2a).

On the other hand, non-endemic representation in Global North collections is evident across most developed countries in Europe and Central Asia, North America and Australia (figure 2b), with some countries like the USA, Great Britain, France and Germany having high values of non-endemic representation ( 71%, 99%, 97% and 99%, respectively). Most Global South countries also showed low non-endemic representation, as their biological collections are primarily composed of native species (figure 2b). Overall, we found an underrepresentation of native fish fauna in most biological collections across the Global South and an overrepresentation of non-endemic name bearers in Global North collections (figure 2c).

### (c) Determinants of per-country number of name bearers and related metrics

**Figure 3.**
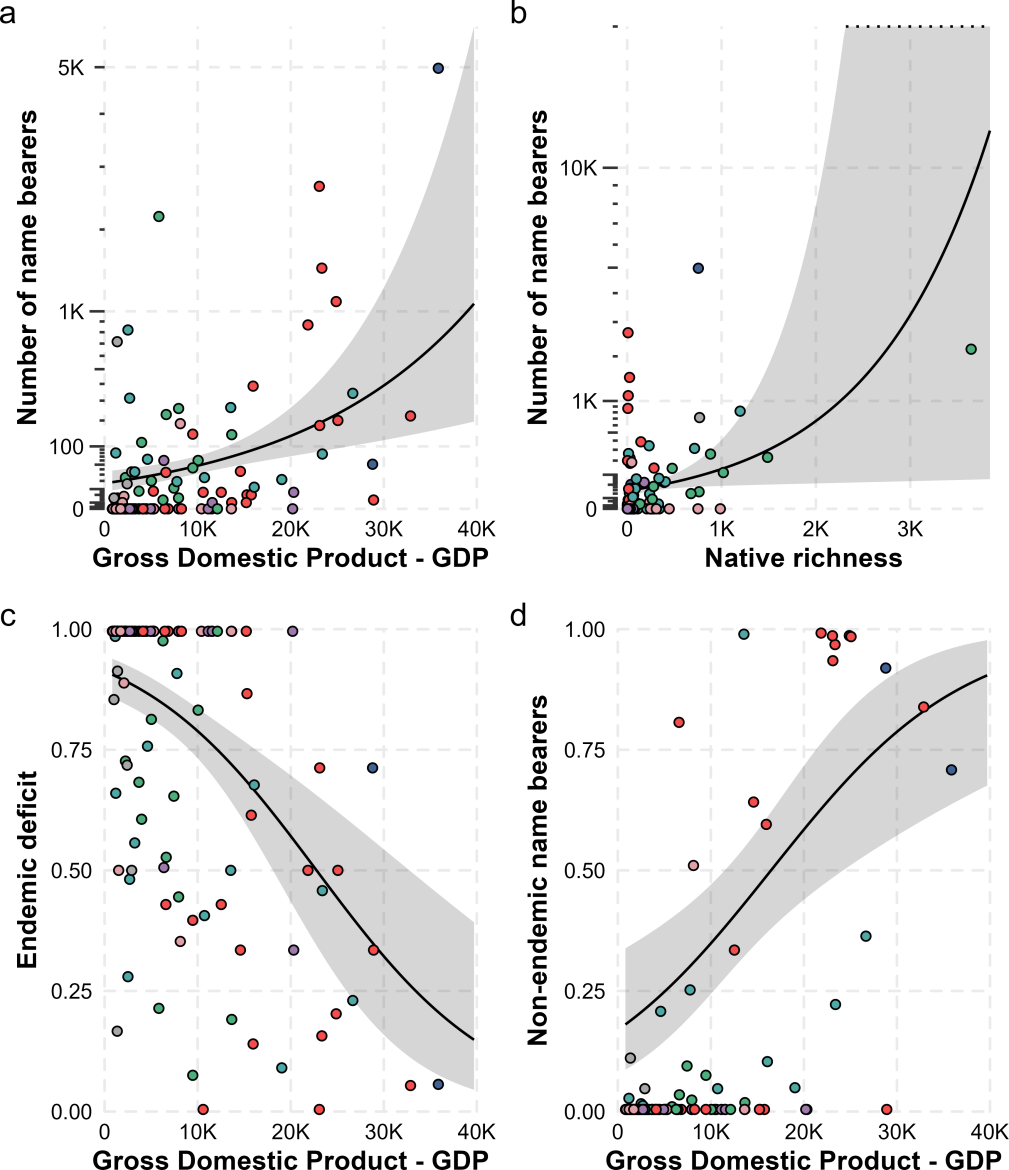
Influence of GDP on countries’ knowledge representation of fish name bearers. The per-country GDP effect on the (a) total number of name bearers, (b) native species richness, (c) endemic deficit and (d) non-endemic representation. All the figures represent the marginal effects of the predictor variables on the response variables. The colours represent the geopolitical regions according to Word Bank. Colour codes for the regions are the same as presented in the inset map in figure 1.

Socio-economic and biological factors strongly shaped concentration, distribution and the characteristics of the per-country name bearers. Specifically, the total number of name bearers within the countries is driven by GDP and native species richness (figure 3a,b).

The characteristics of the country’s biological collections regarding the source of the name bearer (endemic deficit and non-endemic representation) housed in their collections are also explained by GDP. Countries with higher GDP tend to have lower values of endemic deficit in their biological collections than countries with lower GDP. Additionally, wealthier countries (high GDP) often present an overrepresentation of non-endemic name bearers in their biological collections (figure 3c,d). Other factors that we initially hypothesized to be important did not appear to influence our metrics. For example, years since independence did not affect any metric, possibly indicating that, even after independence, little has been done to address other enduring aspects of colonial influence in these countries. Figure 3 represents the most important variables influencing the response variables in our model (except for the total number of name bearers, for which we show the top two variables). Full results of the models, including their parameter estimates, associated standard errors, model adequacy and other models’ statistics, are presented in electronic supplementary material, tables S1–S5.

## 4. Discussion

Despite critiques on the concept of species [27,28] and the possibility of assessing biodiversity patterns using general metrics based on functional, phylogenetic and genetic data, name bearers remain the fundamental unit of nearly all investigations in biodiversity and conservation science [29]. By recognizing name bearers as the cornerstone of biodiversity knowledge, we assume that institutions or countries housing most of the name bearers also possess a disproportionate share of fundamental biological knowledge. While arguments for a more equitable distribution of knowledge could extend beyond ethical grounds and contribute to faster mitigation of biodiversity shortfalls [30], virtually no study has examined country-level asymmetries in the concentration and representation of name bearers worldwide. For the first time, we quantify such biases in reference specimens, which are crucial for the identification and description of new freshwater and brackish fish species, the most diverse vertebrate group on the Earth.

### (a) A glimpse into the biological knowledge accumulation process

Countries with colonial histories and early concentration of economic power have historically accumulated name bearers primarily from tropical countries in Latin America and Africa. This pattern, in which Global South countries serve as providers and Global North countries act as custodians of name bearers, only began to change in the twenty-first century. In recent years, there has been an increase in the retention of specimens in the countries where they were collected. Despite some recent progress, most freshwater fish species discovered in the early stages remain housed in museums located far from their original collection sites. While this situation could foster international cooperation by leveraging materials to advance ecological and conservation knowledge [31], it does not ensure symmetrical collaboration. Global North institutions often retain control over name bearers’ accessibility, perpetuating existing imbalances and limiting access to this material to only a select few researchers (see [32] for a discussion on this topic).

Another critical aspect highlighted by our findings is the continued practice of researchers sending collected specimens back to their home countries, particularly in North America and Europe and Central Asia. Past naturalistic exploration like the Thayer Expedition [33] in Brazil advanced biological collections, but the persistence of this practice indicates that countries with a high concentration of foreign name bearers still overlook the consequences of this accumulation process for inclusivity and the development of biodiversity science. The uneven distribution of name bearers also raises both ethical and legal issues. There is evidence, particularly from palaeontological material, of illegal samplings [11,34]. Even when material is legally exported, ethical issues arise regarding the lack of transparency in how biological material is collected and exported (e.g. parachute science [12]). Protective laws and regulations can help maintain, protect and preserve important biological samples within the country where they were collected. For instance, Brazil implemented such regulations in 1990 [35] mandating that significant biological reference materials (e.g. holotype and syntypes) be retained within the country, either completely or partially in the case of paratypes. This measure has contributed to an increase in name bearer retention in Brazil during the twenty-first century, as evidenced by the substantial portion of name bearers from Brazil shown in figure 1.

### (b) Knowledge split and its consequences for biodiversity research

Regardless of the underlying causes for the biased name bearer distribution, this biodiversity knowledge split (a reference to the habitat split phenomenon [36]) poses additional challenges to Global South researchers in at least three different ways. First, there is a separation of the sampling sites from where validation can occur more precisely. Second, accessing name bearers in foreign countries incurs higher traveling expenses for researchers, particularly those from countries where science has faced significant financial challenges (e.g. Brazil [37] and Colombia [38]) in recent years and decades. Third, relative to domestic standards, the international legislation imposes additional requirements for visiting researchers from the Global South, including more expensive visa fees and lengthier processing times [39]. The alternative is not necessarily simpler, as loans of name bearers often involve additional bureaucracy when specimens must be transported across international borders [39] and carry the risk of damage or destruction during transit [15]. These factors collectively delay the cataloguing and discovery of species, particularly in tropical countries of Global South where biodiversity is still largely unknown [4,40].

The historical growth of Global North collections in both infrastructure and personnel likely contributes to the emergence of the latitudinal taxonomy gradient (LTG) [3], where species in the tropics are less likely to be accurately recognized or classified [29,41,42]. The persistence of uncertainties in tropical species delimitation is closely tied to the unequal distribution of name bearers and preserved specimens, which are disproportionately concentrated in the Global North, far from regions where most species discoveries occur (i.e. tropical regions). The availability of a larger number of preserved specimens increases the chances of ecological, phylogenetic and taxonomic research, which in turn influences species classification and can lead to biased inferences in ecology, evolution and conservation [43–45]. Because of the LTG, tropical regions probably harbour more species than currently estimated, as many tropical species remain hidden within already described taxa. While taxonomic revisions are critical to describe these species, the fact that most of the early-discovered freshwater fishes requiring revisionary studies are located far from their native regions underscores the persistent challenges in addressing biodiversity knowledge shortfall.

### (c) Overcoming barriers to name bearer distribution

Recent proposals have advocated for the use of alternative materials, such as tissues and digitized images to improve the accessibility of museum specimens (e.g. [8,46,47]). Despite the importance of those initiatives, with very successful examples in herbarium collections [48], mitigation of biodiversity knowledge shortfalls will require more than technocratic fixes [30]. The accessibility problem to name bearer material cannot rely solely on ‘remote strategies’, because not all specimens can be easily digitized [7]. Fishes are a good example of how challenging the digitization of specimens can be as it requires high-quality and detailed images of anatomical structures to reach unambiguous species identification [49]. Important diagnostic characters, including internal structures like bones and swimbladders, cannot be captured in digitized images unless in high-quality three-dimensional images generated by, for example, CT scans. Additionally, it is possible that the digitization of natural collections replicates the inequality and the inaccessibility of data created by a colonial past that shaped these collections [50]. Finally, some successful and comprehensive examples of projects that aim to provide high-quality three-dimensional digitalization images, like *oVert* [51], do not prioritize name bearer specimens because they are not easily accessible or because important characteristics are damaged [51].

The ideal scenario would involve returning representative name bearers to the biological collections of the countries where they were collected. However, this requires lengthy bilateral negotiations, even despite instances of illegal collection [11]. It is also worth mentioning the presence of name bearers in private collections, about which we have limited information [17]. Repatriation often demands more than legal evidence; public pressure is also necessary [52]. Moreover, recipient collections must have the infrastructure to maintain the biological material long-term, which may be lacking in some Global South countries [53]. Thus, a better division of labour and costs is needed towards a global goal of describing and protecting biodiversity [54,55]. This approach would prevent delays in improving biodiversity knowledge and its benefits [13]. An alternative would be to facilitate the accessibility of name bearers by covering the costs for researchers from the countries of origin to visit name bearers housed in foreign biological collections [55]. Multilateral organizations, like the Convention on Biological Diversity, could promote research groups to secure funding that would allow researchers working on taxonomic research to visit specimens housed in different museums. We suggest that programmes for visiting research explicitly incorporate aspects of the origin of the material and of the researchers in their evaluations, giving preference for researchers working in research institutes in countries where the material was sampled.

Another important step towards a fairer and more equitable distribution of knowledge involves addressing narratives found in museums and natural history collections. Usually, these narratives highlight the achievements of Western white men, overlooking the historical exploration of the countries from which the material was taken. We agree with Ashby & Machin [56] that including stories about the troubling social histories associated with acquiring natural history specimens is crucial for biological collections and curators to reflect the societies they serve accurately.

One of the most direct benefits of retaining name bearers locally is the advantage for the research community and broader society, as it supports local scientific and educational development. The underrepresentation of name bearers in the Global South has probably decreased investments that could foster local taxonomic science [54], which is now indirectly reflected along latitudinal gaps and biases in biodiversity knowledge [41,55,56]. To illustrate the neglect of this issue, international agreements like the Nagoya Protocol, which aim to ensure fair and equitable sharing of biodiversity benefits, only address genetic resources (e.g. [57]), ignoring the importance of housing reference material for boosting scientific development locally.

## 5. Conclusion

Our results, using fishes as a model organism, reveal an uneven distribution of biological knowledge worldwide, leading to both global and local (country-level) consequences. The knowledge housed by a country often fails to represent its native freshwater and brackish fish fauna accurately. To address these biological knowledge gaps, it is crucial to facilitate access to these materials for researchers from the countries where they were collected. Otherwise, regardless of the available technology and methods for monitoring biodiversity, our understanding of the natural world will remain inequitable. Beyond identifying the issue, we hope this study serves as a catalyst for developing solutions that promote a more equitable and fair distribution of biological knowledge, one that does not replicate the existing economic and social inequalities in our society.

## Data Availability

The primary data used in this article are freely available from Eschemeyer’s CAS [22]. All data and code used to reproduce the figures and models are available in the Zenodo repository [58]. The source code and a reproducible workflow for all analyses can be found at GitHub [59]. The electronic supplementary material provides a detailed overview of the data used. Supplementary material is available online [60].
